# Myocardial infarction risk is increased by periodontal pathobionts: a cross-sectional study

**DOI:** 10.1038/s41598-022-19154-z

**Published:** 2022-11-03

**Authors:** C. Joshi, A. Mezincescu, M. Gunasekara, A. Rudd, H. Botchorichvili, S. Sabir, C. Dospinescu, A. Noman, D. Hogg, G. Cherukara, D. McLernon, K. Hijazi, D. Dawson

**Affiliations:** 1 Institute of Dentistry, School of Medicine, Medical Sciences and Nutrition, University of Aberdeen, Foresterhill, Aberdeen, AB25 2ZR UK; 2 Department of Cardiology and Institute of Dentistry, School of Medicine, University of Aberdeen, Level 1, Aberdeen Royal Infirmary, Foresterhill, Aberdeen, AB25 2ZD UK; 3 Medical Statistics Team, Institute of Applied Health Sciences, School of Medicine, Medical Sciences and Nutrition, University of Aberdeen, Aberdeen, UK

**Keywords:** Atherosclerosis, Microbiology, Risk factors, Cardiovascular diseases, Dental diseases

## Abstract

To establish the role of periodontal pathobionts as a risk factor for myocardial infarction, we examined the contribution of five periodontal pathobionts and their virulence genes’ expressions to myocardial injury (Troponin-I) and coronary artery disease burden (SYNTAX-I scores) using hierarchical linear regression. Pathobiont loads in subgingival-plaques and intra-coronary-thrombi were compared. Troponin-I release increased with one 16S rRNA gene copy/ng DNA of *Porphyromonas gingivalis* (β = 6.8 × 10^–6^, 95% CI = 1.1 × 10^–7^–2.1 × 10^–5^), one-fold increased expressions of *fimA* (β = 14.3, 95% CI = 1.5–27.1), *bioF-3* (β = 7.8, 95% CI = 1.1–12.3), *prtH* (β = 1107.8, 95% CI = 235.6–2451.3), *prtP* (β = 6772.8, 95% CI = 2418.7–11,126.9), *ltxA* (β = 1811.8, 95% CI = 217.1–3840.8), *cdtB* (β = 568.3, 95% CI = 113.4–1250.1), all *p* < 0.05. SYNTAX-I score increased with one 16S rRNA gene copy/ng DNA of *Porphyromonas gingivalis* (β = 3.8 × 10^–9^, 95% CI = 3.6 × 10^–10^-1.8 × 10^–8^), one-fold increased expressions of *fimA* (β = 1.2, 95% CI = 1.1–2.1), *bioF-3* (β = 1.1, 95% CI = 1–5.2), *prtP* (β = 3, 95% CI = 1.3–4.6), *ltxA* (β = 1.5, 95% CI = 1.2–2.5), all *p* < 0.05. Within-subject *Porphyromonas gingivalis* and *Tannerella forsythia* from intra-coronary-thrombi and subgingival-plaques correlated (rho = 0.6, *p* < 0.05). Higher pathobiont load and/or upregulated virulence are risk factors for myocardial infarction.

**Trial registration:** ClinicalTrials.gov Identifier: NCT04719026.

Previous case–control studies have demonstrated an association between suboptimal periodontal health and increased acute myocardial infarction risk^1,2^. Periodontitis develops in a susceptible host due to a disruption of host-microbe homeostasis. Such disruption is precipitated by bacterial species, known as “pathobionts” which colonise periodontal tissue irrespective of disease activity but become pathogenic under certain environmental conditions^3^. Periodontal pathobiont species include *Porphyromonas gingivalis, Tannerella forsythia, Treponema denticola, Aggregatibacter actinomycetemcomitans* and *Prevotella intermedia*^4^*.* Indeed, recent data identified periodontal bacterial DNA in occlusive intra-coronary thrombi of acute myocardial infarction patients, supporting the possibility of bacterial translocation to atherosclerotic plaque^5–7^. Some studies have suggested that periodontitis is linked to coronary artery disease by the direct systemic dissemination of periodontal pathobionts^8–11^. However, the relationship between the load and/or virulence profile of periodontal pathobiont species and the extent of myocardial injury along with coronary artery plaque burden has not been reported. In this study, we sought to investigate if, in addition to clinical markers of periodontitis, periodontal pathobiont colonisation and virulence relate to worse cardiovascular status, namely infarct size and/or increased coronary artery disease burden in a cohort of patients presenting with acute myocardial infarction. In a subset of acute myocardial infarction patients, who presented with ST-segment elevation on the electrocardiogram, we explored whether the same pathobiont species can be detected from both subgingival plaque and coronary thrombus aspirated during the primary percutaneous intervention.

## Results

A total of 863 type-1 myocardial infarction patients were screened between March 2018-September 2020, of which 160 were eligible for this study. Participants’ baseline characteristics are described in Tables 1 and 2. The cohort was representative of the typical myocardial infarction population: higher prevalence of middle-aged men from a spectrum of socio-economic backgrounds, current or former smokers, high body-mass index (BMI), history of hypertension, diabetes, hypercholesterolaemia, as typical comorbidities. Patients had mild leucocytosis with neutrophilia and mildly elevated C-reactive protein levels, typical of systemic pro-inflammatory status associated with acute myocardial infarction. Two-thirds presented with ST-segment elevation on the electrocardiogram. Also, two-thirds of myocardial infarction patients had clinical periodontitis. The clinical periodontal measurements, pathobiont loads or antibody levels did not differ significantly between the patients with ST-segment elevation and non-ST-segment elevation on the electrocardiogram.

**Table 1:** General clinical characteristics of the study population.

	Myocardial infarction population (*n* = 160)
Males, *n* (%)	137 (86)
**Age in years, (median, IQRs)**	60 (54 to 67)
**BMI in kg/m**^2^**, n (%)**	
Obese (≥ 30 kg/m^2^) and overweight (25–29.9 kg/m^2^)	131 (82)
Normal (≤ 24.9 kg/m^2^)	29 (18)
**Scottish index of multiple deprivation (SIMD), n (%)**	
Deprived (quintiles 1 to 3)	62 (39)
Less deprived (quintiles 4 and 5)	98 (61)
**Smoking status, n (%)**	
Current and former smokers	94 (59)
Non-smokers	66 (41)
**Increased alcohol consumption, (%)**	127 (79)
**Hypertension, n (%)**	68 (42)
**Diabetes (type-1 and 2), n (%)**	34 (21)
**Hypercholesterolemia, n (%)**	41 (26)
**Self-reported physical activity, n (%)**	
Sedentary activity	19 (12)
Moderate activity	139 (87)
High activity	2 (1)
**Family history of cardiovascular disease, n (%)**	102 (64)
**Family history of periodontitis, n (%)**	21 (13)
**Prior statin use, n (%)**	41 (26)
**Presenting electrocardiogram, n (%)**	
ST-segment elevation myocardial infarction	94 (59)
Non- ST-segment elevation myocardial infarction	66 (41)
**Echocardiography-derived left ventricular ejection fraction (EF), n (%)**	
Severely impaired (EF < 30%)	6 (4)
Moderately impaired (EF 31–45%)	32 (20)
Mildly impaired (EF 46–54%)	68 (42)
Normal (EF > 55%)	54 (34)
**Pro-bleeding post-MI medications, n (%)**	
Dual anti-platelet therapy	132 (82)
Dual anti-platelet therapy + anticoagulant	28 (18)
**Number of diseased coronary vessels, n (%)**	
Single vessel disease	66 (41)
Two vessel disease	64 (40)
Three vessel disease	30 (19)
**Periodontitis status, n (%)**	
Periodontally-healthy	54 (34)
Periodontitis	106 (66)
Mild periodontitis (Stage I)	4 (2)
Moderate periodontitis (Stage II)	53 (33)
Severe periodontitis (Stage III and IV)	49 (31)

Descriptive categorical measures are expressed as number (*n*) and corresponding percentage (%).

**Table 2:** Biochemical and microbiological characteristics of the study population.

	Myocardial infarction population (*n* = 160)
**Blood biochemistry, (median, IQRs)**	
**White cell count (normal range: 4–10 × 10**^**9**^**/L**)	10.4 (8.5–12.2)
**Neutrophils (normal range: 1.5–7 × 10**^**9**^**/L)**	7.7 (5.6–9.6)
**Lymphocytes (normal range: 1.5–4 × 10**^**9**^**/L)**	1.6 (1.2–2.1)
**Platelets (normal range:140–400 × 10**^**9**^**/L)**	258 (218–303)
**C-reactive protein (normal range: 0–4 mg/L)**	17 (3–43)
**Total cholesterol (normal range: 3.4–5.2 mmol/L)**	4.8 (3.7–5.7)
**Low-density lipoprotein (normal range: 1.4–5 mmol/L)**	2.9 (1.9–3.7)
**12-h Troponin I in ng/L, median (IQRs)**	24,482 (2869–91,983)
**SYNTAX-I score, median (IQRs)**	17 (10–26)
**Periodontal clinical indicators, median (IQRs)**	
PISA** (mm^2^)	330 (190–428)
Mean-Probing Pocket Depth (mm)	1.92 (1.59–2.31)
Mean-Clinical Attachment Loss (mm)	1.99 (1.66–2.43)
Number of missing teeth	6 (3–11)
Percentage plaque index (%)	44 (31–56)
Percentage bleeding index** (%)	45 (37–56)
**Subgingival periodontal pathobiont loads in 16S rRNA gene copies/ng DNA, median (IQRs)**	
*Porphyromonas gingivalis*	2.4 × 10^6^ (4 × 10^4^–9.8 × 10^9^)
*Tannerella forsythia*	1.5 × 10^6^ (2.4 × 10^4^–6.6 × 10^9^)
*Aggregatibacter actinomycetemcomitans*	4.5 × 10^5^ (1.2 × 10^5^–5.1 × 10^6^)
*Prevotella intermedia*	2.8 × 10^5^ (1.9 × 10^4^–5.8 × 10^9^)
**Anti-*P. gingivalis* LPS antibodies in ELISA units (EU), median (IQRs)**	
Serum IgG	16.6 (12.7–19.6)
Serum IgM	9.9 (7.6–12.2)
Serum IgA	9.5 (7.5–12.1)
Salivary IgA2	8.2 (5.6–15.1)

Quantitative measures are expressed as medians and interquartile ranges (IQRs). Periodontitis indicators measured as: site-specific (6 measurements per tooth)—PPD, CAL, BoP; subject-specific- PISA, mean PPD, mean CAL, percentage plaque and bleeding indices. *PISA* periodontal inflamed surface area, *PPD* probing pocket depths, *CAL* clinical attachment loss, *SYNTAX* The Synergy between percutaneous coronary intervention with TAXUS drug-eluting stent and Cardiac Surgery Score, *ELISA* The enzyme-linked immunosorbent assay. ** = values of these variables may be influenced by the antiplatelet therapy given in acute myocardial infarction patients.

### Quantitative indicators of clinical periodontitis and periodontal pathobionts’ load

**Figure 1:**
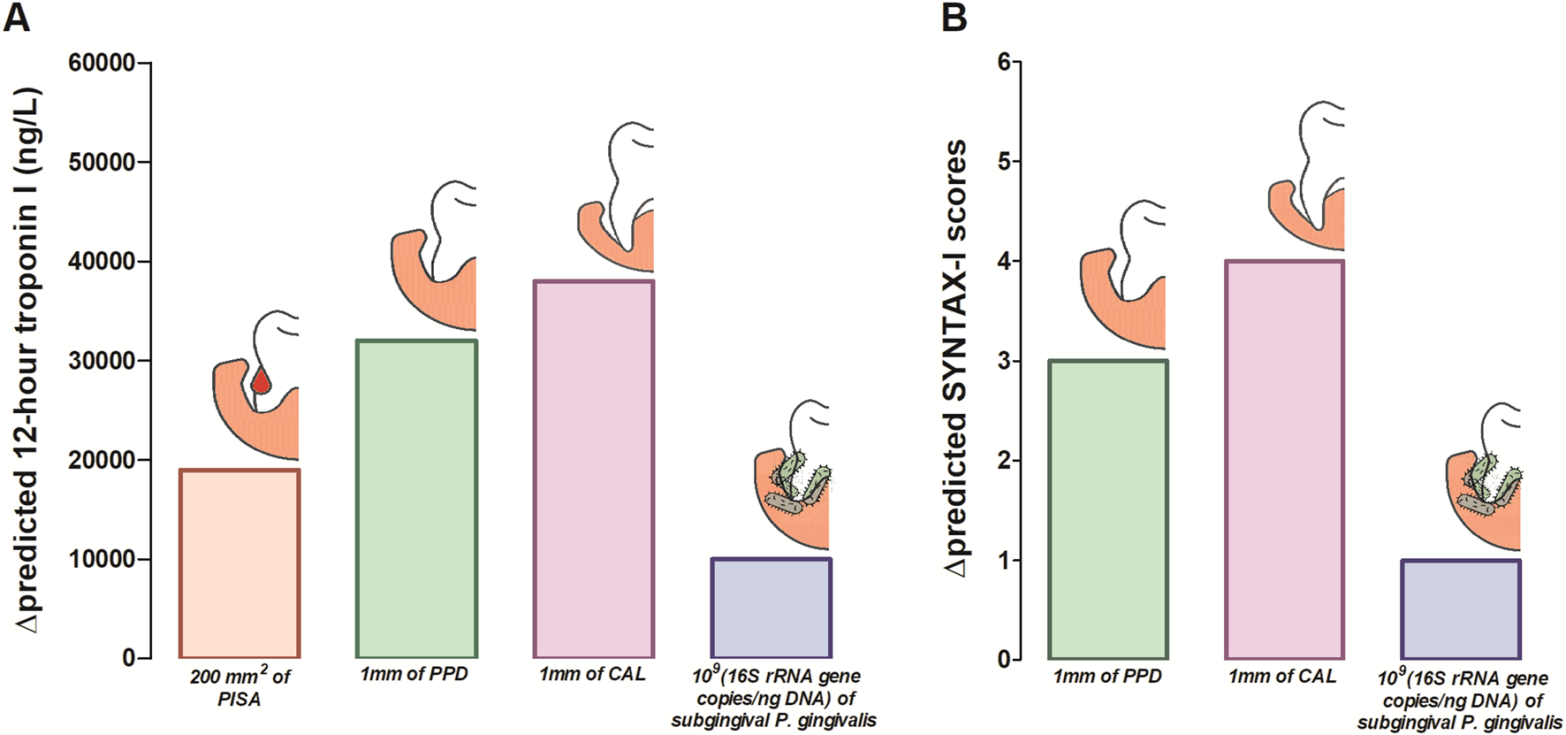
Change in the predicted (**A**) 12-h Troponin I, (**B**) SYNTAX-I scores, with worsening markers of periodontitis and *P. gingivalis* load. (**A**) the incremental effect of three clinical indicators of periodontitis (PISA, mean-PPD, mean-CAL) and subgingival *P. gingivalis* load on 12-h Troponin I levels, (**B**) incremental effect of mean-PPD, mean-CAL and subgingival *P. gingivalis* load on SYNTAX-I score. *PISA* periodontal inflamed surface area, *PPD* probing pocket depths, *CAL* clinical attachment loss.

The basic model-I showed that age, BMI, diabetes, smoking and ST-elevation were significant predictors of myocardial injury extent as judged by troponin (Troponin I) levels (all *p* < 0.05), whereas age, smoking and ST-elevation were predictors of coronary artery disease burden, measured as the SYNTAX-I scores by quantitative angiography (all *p* < 0.05) (Table 3, upper panel). Subsequently (Table 3, lower panel), after adjusting for basic confounders, hierarchical linear regression revealed that there was higher Troponin I release with each 1 mm^2^ periodontal inflamed surface area (PISA) (model II, β = 94.8, 95% CI = 33.2–134.7, *p* = 0.02), 1 mm mean-probing pocket depths (PPD) (model III, β = 32,000.3, 95% CI = 6681.3–51,581.1, *p* = 0.03), 1 mm mean-clinical attachment loss (CAL) (model IV, β = 38,110.4, 95% CI = 9154.5–55,662.1, *p* = 0.01), one 16S rRNA gene copy/ng DNA of *P. gingivalis* (model V, β = 6.8 × 10^–6^, 95% CI = 1.1 × 10^–7^–2.1 × 10^–5^, *p* = 0.01). Similarly, SYNTAX-I scores increased with each 1 mm mean-PPD (model III, β = 3.1, 95% CI = 2.5–4.8, *p* = 0.02), 1 mm mean-CAL (model IV, β = 3.5, 95% CI = 1.1–5.1, *p* = 0.03), one 16S rRNA gene copy/ng DNA of *P. gingivalis* (model V, β = 3.8 × 10^–9^, 95% CI = 3.6 × 10^–10^–1.8 × 10^–8^*, p* = 0.003). The independent addition of PISA had no significant effect on the SYNTAX-I score. The addition of subgingival *T. forsythia, A. actinomycetemcomitans* and *P. intermedia* bacterial loads had no significant effect on Troponin I levels or SYNTAX-I scores. Figure 1 shows the incremental increase in Troponin I levels, and SYNTAX-I scores due to the clinically meaningful changes in PISA, mean-PPD, mean-CAL and *P. gingivalis* bacterial load (rather than unit change as expressed by linear regression).

**Table 3:** Hierarchical linear regression of periodontitis-related indicators with 12-h Troponin I and SYNTAX-I scores as outcome variables (*n* = 160).

Established risk factors (basic model I)	Entire acute MI patient cohort (*n* = 160)					
	12-h Troponin I			SYNTAX-I score		
	β	(95% CI)	*p*	β	(95% CI)	*p*
Age	− 2356.2	(− 4114.1 to − 598.3)	0.001*	0.2	(0.1 to 0.4)	0.007*
Gender (male vs. female)	10,927.4	(− 37,401.6 to 59,257.1)	0.42	1	(− 5.5 to 3.5)	0.66
SIMD quintiles (≤ 3 vs > 3)	− 8496.2	(− 23,279.6 to 6287.2)	0.55	− 0.3	(− 1.6 to 1.1)	0.68
BMI (obese and overweight vs. normal)	23,328.9	(5631.3 to 47,289.2)	0.04*	− 0.4	(− 2.6 to 1.7)	0.69
Diabetes (presence vs absence)	− 9001.3	(− 17,148.1 to − 3715.1)	0.001*	0.6	(− 1.2 to 2.4)	0.53
Smoking (current and former smokers vs non-smokers)	12,655.6	(9350.1 to 34,661.4)	0.02*	1.9	(0.2 to 3.9)	0.03*
ST elevation (ST-elevation vs non-ST-elevation myocardial infarction)	− 108,398.9	(− 142,139.9 to − 74,657.9)	< 0.001*	1.2	(0.3 to 2.8)	0.02*
Adjusted R^2^ for model-I (established risk factors plus ST-elevation)	0.54			0.52		

Subsequent models (II to XII)	12-h Troponin I			SYNTAX-I scores		
Clinical indicators of periodontitis	β	(95% CI)	*p*	β	(95% CI)	*p*
Model II (model I + PISA in mm^2^)	94.8	(33.2 to 134.7)	0.02*	0.003	(− 0.001 to 0.007)	0.13
Model III (model I + mean PPD in mm)	32,000.3	(6681.3 to 51,581.1)	0.03*	3.1	(2.5 to 4.8)	0.02*
Model IV (model I + mean CAL in mm)	38,110.4	(9154.5 to 55,662.1)	0.01*	3.5	(1.1 to 5.1)	0.03*
**Subgingival periodontal pathobiont load (expressed as 16S rRNA gene copies/ng DNA)**						
Model V (model I + *Porphyromonas gingivalis* load)	6.8 × 10^−6^	(1.1 × 10^−7^ to 2.1 × 10^−5^)	0.01*	3.8 × 10^−9^	(3.6 × 10^−10^ to 1.8 × 10^−8^)	0.003*
Model VI (model I + *Tannerella forsythia* load)	1.2 × 10^−8^	(− 6.2 × 10^−2^ to 3 × 10^−6^)	0.7	8.4 × 10^−9^	(− 1.2 × 10^−3^ to 2.9 × 10^−7^)	0.4
Model VII (model I + *Aggregatibacter actinomycetemcomitans* load)	1.5 × 10^−6^	(− 1.5 × 10^−2^ to 3.4 × 10^−4^)	0.1	2.1 × 10^−7^	(− 1.1 × 10^−2^ to 4.3 × 10^−5^)	0.1
Model VIII (model I + *Prevotella intermedia* load)	1.8 × 10^−6^	(− 1.1 × 10^−2^ to 3.2 × 10^−4^)	0.3	1.8 × 10^−7^	(− 1.3 × 10^−2^ to 3.1 × 10^−5^)	0.4
**Anti-*P. gingivalis* LPS antibody isotypes [expressed as ELISA (EU) units]**						
Model IX (model I + serum IgG antibody levels)	− 1158.6	(− 4448.5 to 2131.3)	0.5	0.1	(− 0.1 to 0.4)	0.2
Model X (model I + serum IgM antibody levels)	1432.6	(− 2635.3 to 5500.6)	0.4	0.3	(− 0.1 to 0.6)	0.2
Model XI (model I + serum IgA antibody levels)	6274.3	(− 1843.7 to 10,704.8)	0.3	0.3	(− 0.08 to 0.7)	0.1
Model XII (model I + salivary IgA2 antibody levels)	751.4	(− 1666.1 to 3169)	0.5	0.2	(0.03 to 0.4)	0.1

The established risk factors were introduced in model I followed by individual additions of clinical periodontal disease indicators: per mm^2^ in periodontal inflamed surface area (PISA) (model II), per mm increase mean probing pocket depths (PPD) (model III), and mean clinical attachment loss (CAL) (Model IV), respectively; followed the subgingival increase of 16S rRNA gene copies/ng DNA microbial loads of *P. gingivalis* (Model V), *T. forsythia* (Model VI), *A. actinomycetemcomitans* (Model VII), *P. intermedia* (Model VIII) and then per EU unit increase of *anti-P. gingivalis LPS* antibody isotypes: serum IgG (Model IX), serum IgM (Model X), serum IgA (Model XI), and salivary IgA2 (Model XII). *R*^2^ coefficient of determination, *β* unstandardised regression coefficient, *CI* confidence interval. * = statistically significant (*p* < 0.05), *BMI* body mass index, *SIMD* Scottish Index of. Multiple Deprivation, *SYNTAX* The Synergy between percutaneous coronary intervention with TAXUS drug-eluting stent and Cardiac Surgery Score, *ELISA* The enzyme-linked immunosorbent assay, *LPS* lipopolysaccharide.

### Increased expression of periodontal pathobionts’ virulence genes

In a myocardial infarction subgroup (*n* = 40), hierarchical linear regression models revealed that age, BMI, diabetes, smoking and ST-segment elevation on the electrocardiogram were significant predictors of the extent of myocardial injury (all *p* < 0.05), whereas age, smoking and ST-segment elevation significantly predicted coronary artery disease burden (*p* < 0.05 for both) (Table 4, upper panel).

**Table 4:** Hierarchical linear regression of periodontal pathobionts’ virulence gene expressions with 12-h Troponin I and SYNTAX-I scores (*n* = 40).

Established risk factors (model I)	Subgroup myocardial infarction patient cohort (*n* = 40)					
	12-h Troponin I			SYNTAX-I score		
	β	(95% CI)	*p*	β	(95% CI)	*p*
Age	− 3814.4	(− 7401.2 to − 227.6)	0.02*	0.1	0.08 to 0.2	0.03*
BMI (obese and overweight vs normal)	− 5239	(− 13,132.1 to − 2781.8)	0.02*	0.2	− 4 to 4.3	0.94
Diabetes (presence vs absence)	1254.1	(829.3 to 16,591.4)	0.01*	4.2	− 1.7 to 10.1	0.51
Smoking (current and former smokers vs non-smokers)	19,820.6	(1941.8 to 25,231.9)	0.04*	− 4.4	− 7.9 to − 1.6	0.02*
ST elevation (ST-elevation vs non-ST-elevation myocardial infarction)	116,030.1	(42,959.6 to 189,100.7)	0.003*	6.3	1 to 11.8	0.02*
Adjusted R^2^ for model-I (established risk factors plus ST-elevation)	0.54			0.49		

Subsequent models (II to XIV)	12-h Troponin I			SYNTAX-I scores		
Expression of virulence genes (expressed as fold change)						
*Porphyromonas gingivalis*	β	(95% CI)	*P*	β	(95% CI)	*p*
Model II (model I + *kgp*) (upregulated in 16 patients out of 40)	1.7	(− 0.7 to 4.1)	0.2	0.02	(− 0.001 to 0.1)	0.2
Model III (model I + *rgpA*) (upregulated in 16 patients out of 40)	2.2	(− 0.2 to 4.5)	0.7	0.01	(− 0.001 to 0.1)	0.1
Model IV (model I + *fimA*) (upregulated in 10 patients out of 40)	14.3	(1.5 to 27.1)	0.002*	1.2	(1.1 to 2.1)	0.002*
Model V (model I + *bioF-3*) (upregulated in 12 patients out of 40)	7.8	(1.1 to 12.3)	0.002*	1.1	(1 to 5.2)	0.005*
***Tannerella forsythia***						
Model VI (model I + *prtH*) (upregulated in 15 patients out of 40)	1107.8	(235.6 to 2451.3)	0.006*	0.003	(− 0.01 to 0.01)	0.7
Model VII (model I + *bspA*) (upregulated in 11 patients out of 40)	22.1	(− 58.2 to 102.2)	0.6	0.04	(− 0.005 to 0.09)	0.4
Model VIII (model I + *siaHI*) (upregulated in 17 patients out of 40)	39.9	(− 36.5 to 116.4)	0.3	0.09	(− 0.04 to 0.22)	0.3
***Treponema denticola***						
Model IX (model I + *prtP*) (upregulated in 14 patients out of 40)	6772.8	(2418.7 to 11,126.9)	0.002*	3	(1.3 to 4.6)	0.001*
Model X (model I + *msp*) (upregulated in 8 patients out of 40)	322.5	(− 310.5 to 955.6)	0.3	0.3	(− 0.1 to 0.7)	0.9
***Aggregatibacter actinomycetemcomitans***						
Model XI (model I + *ltxA*) (upregulated in 4 patients out of 40)	1811.8	(217.1 to 3840.8)	0.007*	1.5	(1.2 to 2.5)	0.01*
Model XII (model I + *cdtB*) (upregulated in 3 patients out of 40)	568.3	(113.4 to 1250.1)	0.009*	0.002	(− 0.005 to 0.01)	0.5
***Prevotella intermedia***						
Model XIII (model I + *clpB*) (upregulated in 10 patients out of 40)	− 54.1	(− 331.5 to 223.2)	0.8	0.04	(− 0.11 to 0.09)	0.9
Model XIV (model I + *dnaK*) (upregulated in 13 patients out of 40)	− 7.2	(− 1730.3 to 1715.8)	0.9	0.004	(− 0.01 to 0.02)	0.64

The established common risk factors were introduced in model I followed by individual additions of per unit fold increase in expression levels of *P. gingivalis*-related virulence genes: *kgp* (model II), *rgpA* (model III), *fimA* (model IV), *bioF-3* (model V); followed by *T. forsythia-*related virulence genes*: prtH* (model VI), *bspA* (model VII), *siaHI* (model VIII); followed by *T. denticola*-related virulence genes: *prtP* (model IX), *msp* (model X); followed by *A. actinomycetemcomitans-*related virulence genes: *ltxA* (model XI), *cdtB* (Model XII); followed by *P. intermedia*-clpB (model XIII), dnaK (model XIV). *R*^2^ coefficient of determination, *β* unstandardised regression coefficient, *CI* confidence interval. * = statistically significant (*p* < 0.05), *BMI* body mass index, *SIMD* Scottish Index of. Multiple Deprivation, *SYNTAX* The Synergy between percutaneous coronary intervention with TAXUS drug-eluting stent and Cardiac Surgery Score.

The subsequent models (Table 4, lower panel) showed that myocardial injury (Troponin I release) increased incrementally for every one-fold increase in expression of *fimA* (model IV, β = 14.3, 95% CI = 1.5–27.1, *p* = 0.002), *bioF-3* (model V, β = 7.8, 95% CI = 1.1–12.3, *p* = 0.002), *prtH* (model VI, β = 1107.8, 95% CI = 235.6–2451.3, *p* = 0.006), *prtP* (model IX, β = 6772.8, 95% CI = 2418.7–11,126.9*, p* = 0.002), *ltxA* (model XI, β = 1811.8, 95% CI = 217.1–3840.8, *p* = 0.007), *cdtB* (model XII, β = 568.3, 95% CI = 113.4–1250.1, *p* = 0.009). Similarly, coronary artery disease burden (SYNTAX-I score) worsened after independent addition of one-fold increase in expression of *fimA* (model IV, β = 1.2, 95% CI = 1.1–2.1, *p* = 0.002), *bioF-3* (model V, β = 1.1, 95% CI = 1–5.2, *p* = 0.005), *prtP* (model IX, β = 3, 95% CI = 1.3–4.6, *p* = 0.001), *ltxA* (model XI, β = 1.5, 95% CI = 1.2–2.5, *p* = 0.01).

### Simultaneous detection of periodontal pathobionts in subgingival plaque and intra-coronary thrombi

**Figure 2:**
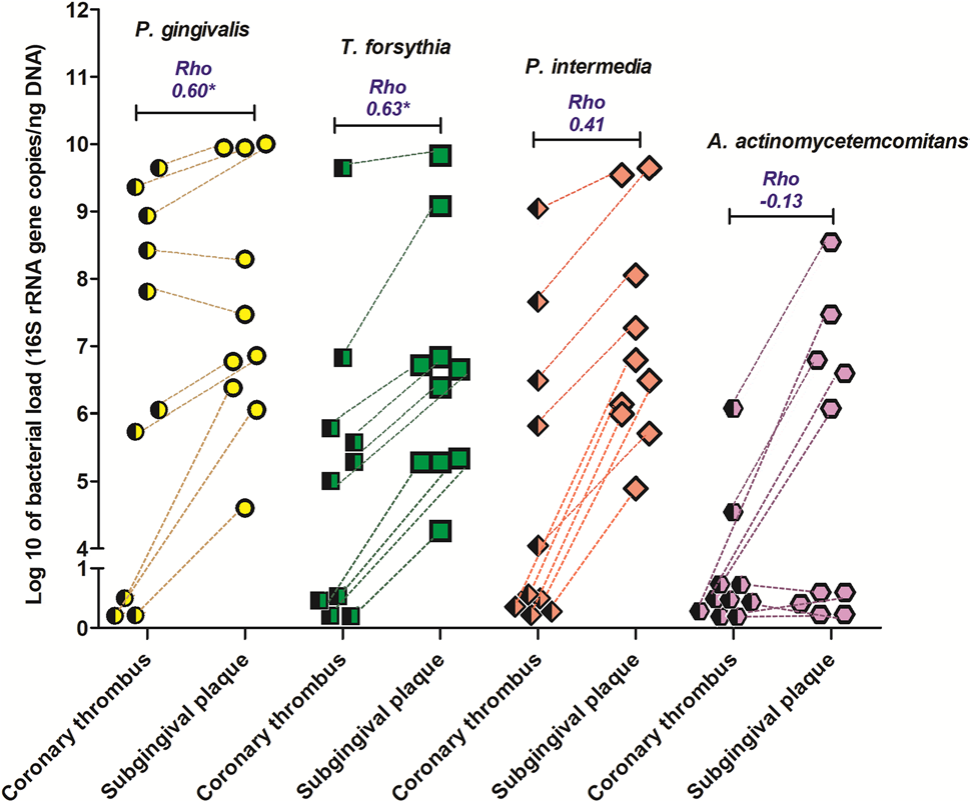
Comparison of within-subject periodontal pathobiont loads in intra-coronary thrombi and subgingival plaques collected from the myocardial infarction patients (*n* = 10). Corresponding bacterial loads (16S rRNA gene copies/ng DNA) of four periodontal pathobionts: *P. gingivalis* (yellow), *T. forsythia* (green), *P. intermedia* (red) and *A. actinomycetemcomitans* (purple) in aspirated intra-coronary thrombi and subgingival plaque samples from ten ST-segment elevation myocardial infarction patients (dashed lines show paired samples for each patient). Rho-spearman’s correlation coefficient, * = *p* < 0.05.

In a subgroup of ten patients with acute ST-segment elevation myocardial infarction who had coronary thrombectomy, three patients were periodontally-healthy. In these three patients, the DNA of four periodontal pathobiont species (*P. gingivalis, T. forsythia, P. intermedia, A. actinomycetemcomitans*) could not be detected in coronary thrombi retrieved during percutaneous coronary intervention (> 35 Cq value). The remaining seven patients had clinical periodontitis of varying severity. The frequency of periodontal pathobiont species detection in their coronary thrombus was highest for *P. gingivalis* (7/10)*,* followed by *T. forsythia* (6/10) and *P. intermedia* (5/10) and lowest for *A. actinomycetemcomitans* (2/10). The comparison of periodontal pathobiont loads in aspirated coronary thrombi and subgingival plaque is shown in Fig. 2. A moderate correlation was observed between bacterial loads detected in coronary thrombi and corresponding subgingival plaque from the same patient for *P. gingivalis* and *T. forsythia* (Spearman’s rho = 0.6 and *p* < 0.05, for both), but not for *P. intermedia or A. actinomycetemcomitans*. No bacterial DNA was detected in percutaneous coronary intervention balloons or venous blood samples. The absolute quantification of *Treponema denticola* was not possible due to the unavailability of genomic DNA of a reference strain.

### Local and systemic immune host profile against Porphyromonas gingivalis

No significant relationship was observed between any of the serum and salivary anti-*P. gingivalis*-LPS antibody isotypes detected by the enzyme-linked immunosorbent assay (ELISA) assays and cardiac outcome measures (12-h Troponin I and SYNTAX-I) (models IX-XII) (Table 3, lower panel).

## Discussion

In a cross-sectional study of acute myocardial infarction patients with varying degrees of clinical periodontitis, we showed that: (1) clinical indicators of active periodontitis (PISA, CAL, PPD), increased abundance of subgingival *P. gingivalis*, and upregulation of virulence genes expressed by the periodontal pathobionts are risk factors for worse cardiac outcomes, namely acute myocardial injury and underlying coronary artery disease burden; (2) simultaneous presence of *P. gingivalis* and *T. forsythia* DNA in intra-coronary thrombi and subgingival plaque at individual patient level. Taken together, these findings demonstrate that in addition to clinical variables, the abundance and virulence of periodontal pathobionts are important factors in the associative relationship between periodontitis and myocardial infarction. Interventional studies are warranted to explore if periodontitis treatment has a beneficial effect in reducing cardiovascular events.

### Study rationale

Despite major advances in primary and secondary prevention through risk factor modification, myocardial infarction remains responsible for most deaths worldwide. Identification of additional contributory risk factors may provide further risk reduction. Periodontitis is one such suggested risk factor. In this study, we evaluated the impact of periodontal pathobiont load and virulence on myocardial infarction risk.

### Periodontitis: prevalence, case definitions, and methodological variations

The periodontitis prevalence in our myocardial infarction patients was higher than in previous studies^1,2^. Whilst dental care differs between countries, there are also differences in the use of periodontitis case definitions and periodontal examination methods. For example, Ryden *et a*l. based their diagnosis on the percentage of radiographic alveolar bone loss^2^, while Gomes-Filho et al., used an in-house case definition^1^. Radiographic examinations are ideally suited for large throughput studies. In this study, we used the latest periodontitis case-definition^12^ as we were able to apply detailed examination in our chosen sample size. To address the suboptimal precision and accuracy of manual periodontal probes, we used a computerised Florida periodontal probe. The Florida probe imparts a constant probing pressure maximising the reliability of clinical periodontal measurements^13^.

### Contribution of clinical indicators of periodontitis, subgingival periodontal pathobiont load and expression of their virulence genes

The collective contribution of inflammatory processes occurring in the periodontal milieu was assessed by three quantitative clinical indicators of periodontitis (PISA, PPD and CAL) as done by the previous investigators^1^. All three significantly predicted the extent of acute myocardial injury, while PPD and CAL were significant markers for underlying chronic coronary artery disease burden. The cumulative marker of periodontal inflammatory burden (PISA) was associated only with acute cardiac outcomes (Troponin I). PISA includes bleeding on probing (BoP) in addition to the other three markers of periodontal disease. Increased periodontal bleeding may be a reflection of the generalised low-grade inflammation present during an acute coronary event. Previous reports suggest that patients on dual antiplatelet therapy generally have no increased periodontal bleeding compared to controls^14^.

Active periodontitis has been long associated with the presence of ‘red complex bacteria’ (*P. gingivalis, T. forsythia, T. denticola)*. These species as well as two other pathobiont species, widely implicated in periodontitis, were the focus of our study (*A. actinomycetemcomitans* and *P. intermedia)*^3,4^. Among the five tested pathobiont species isolated from periodontal pockets, we showed that *P. gingivalis* load was an independent marker of both worse myocardial injury after acute myocardial infarction and higher/more severe chronic plaque burden. However, the presence of high *P. gingivalis* load or other pathobionts per se is not causative of inflammatory disease (locally and systemically), unless accompanied by gene expression of virulence factors^9^. Overexpression of virulence factors underpins the transition of periodontal bacteria from commensals to pathogenic status, driving tissue destruction through proteolytic activity, inflammatory responses, and immune evasion. Animal studies based on ApoE knock-out mice have also suggested a role for these genes in pro-inflammatory responses, leading to atherosclerosis^8^.

For the first time, we demonstrate that the upregulation of genes encoding pathogenic phenotypes in four periodontal bacteria were independent markers of both the extent of myocardial injury and severity of underlying coronary artery disease burden. More specifically, genes implicated are responsible for a range of phenotypes relevant to atherosclerosis, namely: (i) mounting pro-inflammatory responses via TLR-2 pathway (*bioF-3* gene; *P. gingivalis*)^15^, (ii) bacterial adhesion underpinning intracellular and transepithelial invasion (*fimA* gene; *P. gingivalis*)^15^, (iii) degradation of intercellular adhesins facilitating bacterial invasion beyond the epi/endothelium barrier (*prtH* and *prtP* genes; *T. forsythia, T. denticola*)^16,17^, and (iv) host-defence evasion (*ltxA* and *cdtB* genes; *A. actinomycetemcomitans*)^18^. Future studies investigating mechanistic links between these factors and coronary artery disease are warranted.

### Simultaneous detection of periodontal pathobionts in subgingival plaque and intra-coronary thrombi

Periodontal bacteria breach the confines of periodontal pockets using a repertoire of virulence factors. Here we recovered periodontal bacterial DNA in aspirated intra-coronary thrombi responsible for acute myocardial infarction, in keeping with another recent report^5^. However, for the first time, we showed simultaneous recovery of these pathobionts in both subgingival plaque and coronary thrombi, in patients with clinical evidence of periodontitis. The bacterial load detected in coronary thrombi was generally lower than that in subgingival plaque, but in two patients, values were comparable.

Venous blood or coronary stent deployment balloons were negative for periodontal pathobiont DNA, in keeping with the previous findings^5^. This is expected as low-grade systemic bacteraemia, typically associated with daily oral activities, is short-lived and clears rapidly^19^. These findings should be interpreted in the context of the relatively small sample size.

## Limitations

The findings of this study are based on a cross-sectional study design that precludes the deduction of causality or temporal relationship between the analysed variables. Nonetheless, reverse causality supporting the hypothesis that worse acute myocardial injury and/or coronary artery disease burden is increased by clinical and microbial periodontal markers is plausible.

## Conclusion

We demonstrate that in addition to clinical indicators of periodontitis severity, an increased load of subgingival pathobionts and overexpression of their virulence factors are additional markers of increased acute myocardial injury and severity of underlying coronary artery plaque burden. Periodontal pathobiont DNA simultaneous recovery from intra-coronary thrombi and subgingival plaque of patients presenting with acute myocardial infarction supports the concept of bacterial translocation from the oral cavity. These data strengthen the role of periodontitis and periodontal pathobiont species as possible risk factors for myocardial infarction.

## Methods

### Study population

Consecutively consenting eligible patients hospitalised with type-1 myocardial infarction^20^ at Aberdeen Royal Infirmary between March 2018 and September 2020 were enrolled in this single centre, cross-sectional investigation. All those with recent infection, inflammatory or allergic conditions, renal or hepatic impairment, malignancy, pregnant women, or anyone with prior use of antibiotics (past 6 months), anti-inflammatories, calcium-channel blockers, phenytoin or cyclosporin were excluded. Previous studies reported a modest impact of periodontitis on myocardial infarction (coefficient of determination/R^2^ = 0.2)^21^. To investigate the anticipated small effect size in linear regression, a sample size of 130 is required at the power of 80% and alpha of 5% (0.05) (Statistics calculators, v1, 2017, https://www.statskingdom.com/about.html).

### Study protocol

**Figure 3:**
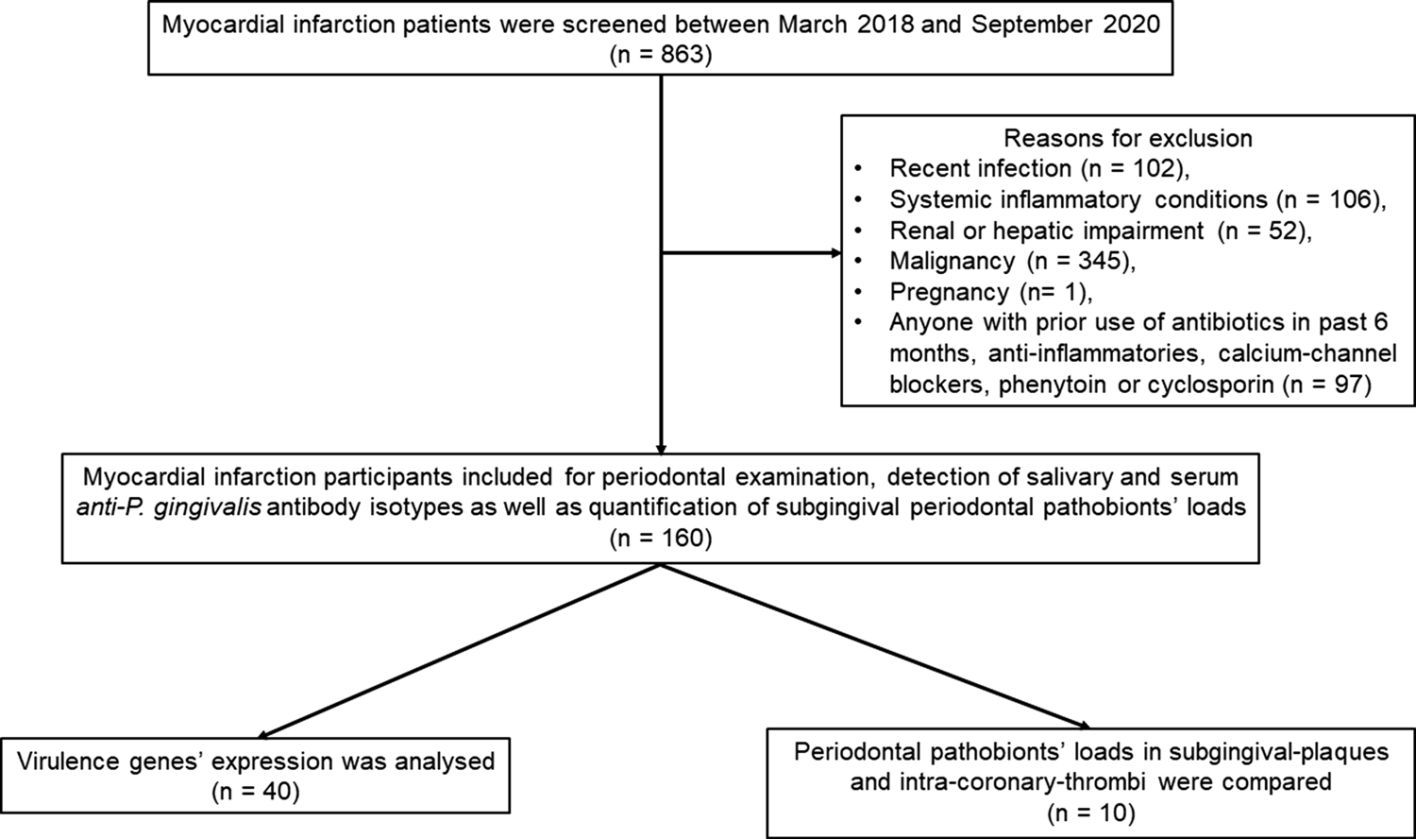
Study flow-chart (STROBE-type diagram).

Within 3 days of myocardial infarction presentation, 160 patients underwent periodontal examination, sample collection (subgingival plaque, saliva, venous blood) in addition to routine collection of 12-h Troponin I (Troponin I) and coronary angiography data. In a sub-group of patients presenting with ST-elevation (*n* = 10), aspirated coronary thrombi and balloons used for coronary stent deployment were collected. This study complies with the Declaration of Helsinki and was approved by the Cambridge East Research Ethics Committee (16/EE/0283, dated 10th May 2016). The study is registered at ClinicalTrials.gov (NCT04719026, dated 22nd January 2021). Written informed consent was obtained directly from all participants according to the World Health Organisation guidelines for good research practice. The Strengthening the Reporting of Observational Studies in Epidemiology (STROBE) guidelines^22^ for reporting cross-sectional studies were followed (Fig. 3) and the checklist is described in the Supplementary file, Fig. 1.

### Periodontal examination

A full periodontal assessment was carried out following the British Society of Periodontology guidelines^23^: gingival recession, probing pocket depth (PPD) and clinical attachment loss (CAL) in millimetres, presence of bleeding-on-probing and dental plaque around six sites (disto-buccal, mid-buccal, mesio-buccal, disto-lingual, mid-lingual, mesio-lingual) were recorded for each tooth; third molars, dental implants and retained roots were excluded. The site-specific measurements (measurements at 6 sites/tooth) were used to calculate mean-PPD, mean-CAL, percentage bleeding^24^ and plaque indices^25^ for each patient. Periodontal Inflamed Surface Area (PISA)^26^, which quantifies bleeding-on-probing-positive periodontal pocket epithelium surface area in mm^2^, was calculated for each patient. A diagram of periodontal measurements is shown in the Supplementary file, Fig. 2. The number of missing teeth was recorded. Periodontitis was diagnosed according to the 2017 definition^12^. A single examiner (CJ), trained and calibrated according to the British Society of Periodontology guidelines, carried out the clinical periodontal examination using a computerised periodontal Florida Probe (Florida Probe Corporation, Florida, USA). Intra-examiner reliability was excellent: intraclass correlation coefficient of 0.8 for both PPD and CAL, performed in ten age/gender-matched controls twice on two consecutive days, part of a larger healthy control population (data not shown).

### Subgingival plaque

In each oral quadrant, one deepest periodontal pocket was selected, dried and isolated using sterile cotton rolls. A sterile ProTaper absorbent paper point X3 (Dentsply Sirona, Surrey, UK) was inserted apically in each periodontal pocket for 30 s and discarded if any bleeding was visually detected. The four paper points were pooled in 200 µL of phosphate-buffered saline (PBS) and stored at − 80 °C. For gene expression assays, an additional pooled sample was collected from the second deepest periodontal pocket/oral quadrant of every fourth recruit.

### Saliva sample

Unstimulated saliva (2 ml) was collected in the morning after 1 h fast (before periodontal examination) using a passive drool method^27^ and stored at − 80 °C.

### Aspirated coronary artery thrombi and percutaneous coronary intervention balloons

When clinically judged appropriate by an interventional cardiologist, a high thrombus load was aspirated in aseptic conditions before primary percutaneous stenting using a Pronto V3 or Export extraction catheter, along with percutaneous coronary intervention balloons (*n* = 10 each). Samples were transferred into sterile microtubes containing PBS and stored at − 80 °C. A venous blood sample from the same patient was used as a comparator.

### Blood sampling

The serum of 10 mL venous blood (*n* = 160) was separated by centrifugation (3000 rpm, 10 min, 4° C), aliquoted and frozen at − 80° C.

### Quantification of primary periodontal pathobionts

Genomic bacterial DNA from subgingival plaque (*n* = 160), aspirated intra-coronary thrombi, respective percutaneous coronary intervention-balloons and venous blood (*n* = 10 each) was extracted using GenElute Kit (Sigma-Aldrich, Dorset, UK). Primers and probes were targeted to the 16S ribosomal RNA gene hypervariable regions (V) of *P. gingivalis* (V3)*, T. forsythia* (V4), *A. actinomycetemcomitans* (V2) *and P. intermedia (*V7) (Supplementary Table 1). Supplementary file, Sect. 1 describes the full quantitative polymerase chain reaction (qPCR) protocol, where bacterial loads were quantified using absolute quantification and reported as 16S rRNA gene copies/ng DNA^28^. The sample positivity for bacterial detection was set at the 35th quantification cycle (Cq) (Supplementary file, Fig. 3).

### Virulence genes expression

Total bacterial RNA was extracted from the additional subgingival plaque samples (*n* = 40) using GenElute Total RNA Purification Kit (Sigma-Aldrich, Dorset, UK) and treated with DNase (Sigma-Aldrich, Dorset, UK). Purified RNA was used as template in one-step quantitative reverse transcription PCR (RT-qPCR) to target thirteen recognised genes encoding major virulence factors of *P. gingivalis (kgp, rgpA, fimA, bioF-3)*^15^; *T. forsythia (prtH, bspA, siaHI)*^17^; *T. denticola (prtP, msp)*^16^; *A. actinomycetemcomitans (ltxA, cdtB)*^18^ *and P. intermedia (clpB, dnaK)*^29^ (Supplementary file, Sect. 2). The biological functions of virulence genes and respective primer–probe sequences are described in Supplementary Tables 2 and 3, respectively. Virulence gene expression was quantified using the ΔΔCT method^30^ against three reference genes (*recA*, *glyA* and *groL)* and expressed as fold change according to ‘the minimum information for publication of quantitative real-time PCR experiments (MIQE)’^31^.

### Local and systemic host immune profile against *Porphyromonas gingivalis*

Serum IgG, IgM, IgA and salivary secretory IgA2 isotype antibody responses against *P. gingivalis* lipopolysaccharide (LPS) were quantified using an enzyme-linked immunosorbent assay (ELISA) (Supplementary file, Sect. 3).

### Cardiac biomarkers and Coronary angiography

Troponin I (TnI) (Siemens ADVIA Centaur TnI Ultra, cut-off 40 ng/L) was collected 12 h from the onset of chest pain and used as a surrogate marker of acute myocardial injury^32^. The invasive coronary angiography study performed during index admission was analysed using quantitative coronary arteriography analysis (QCA) by an investigator (MG) blinded to periodontal status. The Synergy between percutaneous coronary intervention with TAXUS drug-eluting stent and Cardiac Surgery Score (SYNTAX-I, http://www.syntaxscore.com/)^33^ was calculated to quantify the complexity and severity of coronary artery disease burden in native coronary vessels).

### Statistical analyses

Normally distributed variables are shown as mean and standard deviation (SD), skewed variables as median and interquartile ranges (IQR). Categorical variables are summarised as frequencies (n) and percentages (%). The relationship of clinical, microbial, and immunological periodontal indicators on outcome measures (12-h Troponin I and SYNTAX-I scores) was analysed by hierarchical linear regression. Baseline risk factors *shared between periodontitis and myocardial infarction,* such as age, gender, Scottish Index of Multiple Deprivation (SIMD), body mass index (BMI), diabetes, and smoking were built into the basic regression model-I. To account for myocardial infarction type (ST-segment elevation vs non- ST-segment elevation), basic model-I was adjusted for ST-elevation status. Subsequent models were constructed by adding one marker of interest in each model (PISA, mean PPD, mean CAL, subgingival pathobiont loads and antibody levels). Since virulence gene expression levels were analysed in a subgroup (*n* = 40), to reduce the risk of overfitting, a parsimonious basic model-I was constructed with the most important myocardial infarction confounders based on the initial hierarchical linear regression findings i.e., age, BMI, diabetes, smoking and ST-elevation. Each linear regression model provided the incremental change (β) and 95% confidence intervals for acute myocardial injury (12-h Troponin I) or coronary artery disease burden (SYNTAX-I score) brought by a per-unit change in PISA, mean-PPD, mean-CAL, bacterial loads and virulence gene expression, respectively. Periodontal bacterial loads from intra-coronary thrombi and subgingival plaque from the same individuals were compared by Spearman’s correlation. Intra-examiner reliability with clinical periodontal markers was calculated as intraclass correlation coefficient in 10 healthy controls who underwent periodontal examination twice, on consecutive days. Data were analysed using IBM SPSS Statistics v.25 (IBM, Hampshire, UK) and graphs prepared using GraphPad Prism v5.04 (GraphPad Software, San Diego, USA). Statistical significance was set at 5%.

### Conference prize

This work was the winner of the Sir Wilfred Fish Prize at the British Society of Periodontology annual conference, London, November 2021 (https://www.bsperio.org.uk/professionals/awards-prizes-2021).

## Supplementary Information

Supplementary Information.

## Data Availability

The corresponding author has full access to all the datasets used for analysis and it will be shared at a reasonable request.
