# Supplementary material for: Myocardial infarction risk is increased by periodontal pathobionts: a cross-sectional study
Source: Sci Rep. 2022 Nov 3;12:18608. doi: 10.1038/s41598-022-19154-z (PMC9633823; doi:10.1038/s41598-022-19154-z)
Supplement: Supplementary file 1 — Supplementary Information. [file 41598_2022_19154_MOESM1_ESM.pdf]

Supplementary Information file

Title: Myocardial infarction risk is increased by periodontal pathobionts: a cross-sectional study

Brief Title: Periodontal pathobionts, a novel risk factor for myocardial infarction

Joshi C[a], PhD, MDS, LDS RCS (Eng); Mezincescu A[b], PhD, MD MRCP; Gunasekara M[b], MD MRCP; Rudd A[b], RDCS, MSc; Botchorichvili H[a], BSc, MSc; Sabir S[a], BSc, PhD; Dospinescu C[b], PhD, MD MRCP; Noman A[b], PhD, MD MRCP; Hogg D[b], MD FRCP; Cherukara G[a], BDS, PhD, FDS (Rest Dent) RCPS (Glasg); McLernon D[c], PhD; Hijazi K[a], DDS, PhD, MFDS RCPS (Glasg); Dawson D[b], D Phil, MD, FRCP

Dr Hijazi and Dr Dawson contributed equally to this work.

a. Institute of Dentistry, School of Medicine, Medical Sciences and Nutrition, University of Aberdeen, United Kingdom
b. Department of Cardiology and Institute of Dentistry, School of Medicine, University of Aberdeen, Aberdeen, United Kingdom
c. Medical Statistics Team, Institute of Applied Health Sciences, School of Medicine, Medical Sciences & Nutrition, University of Aberdeen, Aberdeen, United Kingdom

Address for Correspondence:

Karolin Hijazi, Institute of Dentistry, AB25 2ZR, Foresterhill, University of Aberdeen, UK

Email: k.hijazi@abdn.ac.uk, ORCID ID: https://orcid.org/0000-0002-8589-2129

Dana Dawson, Cardiology Department, Level 1, Aberdeen Royal Infirmary, AB25 2ZD, Foresterhill, University of Aberdeen, UK

Email: dana.dawson@abdn.ac.uk, ORCID ID: https://orcid.org/0000-0003-2815-4469

Section 1: Quantitative polymerase chain reaction (qPCR) for detection and quantification of periodontal bacterial load

Quantitative polymerase chain reaction assays were conducted on genomic bacterial DNA purified from all clinical samples. All samples were analysed in triplicate using 384 well plates (Sigma-Aldrich, Dorset, UK). Each well contained a 10 µL reaction mixture that included: 5 µL of 2 × Takyon qPCR dTTP MasterMix (Eurogentec, Hampshire, UK), 1 µL of template genomic DNA, 0.5 µL of forward and reverse primers each, 0.2 µL of the probe (Eurogentec, Hampshire, UK) and 2.8 µL nuclease-free water. The primer and probe sequences are listed in **Supplementary Table 1**. The primer (500 nM) and probe (100 nM) concentrations were optimised in a pilot assay (data not shown). In each plate, genomic DNA of *P. gingivalis* 33277 was used as a positive control and nuclease-free water (Sigma-Aldrich, Dorset, UK) as a negative control. All the qPCR assays were performed on a LightCycler 480 Real-Time PCR (Roche Molecular Systems, West Sussex, UK) device. The thermocycling program was set for: 10min at 95 °C (initial denaturation), 40 cycles of 30 s at 95 °C, 1 min at 60 °C. The increase in fluorescence was monitored during PCR amplification and all data was analysed using LightCycler® 480 Software (Roche Molecular Systems, West Sussex, UK). The absolute quantification method [28] was utilised to quantify the bacterial load of all selected periodontal bacterial species. *P. gingivalis* (ATCC 33277), a previously characterised *T. forsythia* isolate, *A. actinomycetemcomitans* isolated from a periodontitis patient at Aberdeen Dental Hospital and *P. intermedia* (ATCC 25611) were used as reference strains to construct the respective standard curves (**Supplementary Figure 3**).

Section 2: Reverse transcription PCR (RT-qPCR) for quantification of the expression of virulence genes

RT-PCR assays were conducted on purified RNA extracted from subgingival plaque and tested in triplicate. The expression levels of three genes per PCR-well were multiplexed using a combination of three dual labelled probes as follows: i) channel 1: 6 FAM and Eclipse Dark Quencher, ii) channel-2: HEX and Eclipse Dark Quencher, iii) channel-3: Cy5 and DDQII. Each PCR-well contained: 5 µL of 2× Takyon qPCR dTTP MasterMix (Eurogentec, Hampshire, UK), 0.25 µL of forward and reverse primers of 3 distinct target genes, respectively; 0.1 µL of probes of 3 distinct target genes, respectively; 1 µL of the template (RNA), 0.1 µL of Takyon One-Step Kit Converter (Eurogentec, Hampshire, UK) and 2.1 µL of nuclease-free water. The primer and probe sequences targeting virulence genes are listed in **Supplementary Table 3.** The concentrations of primers (500 nM) and probes (100 nM) were optimised in a pilot assay (data not shown). Each plate contained purified *P. gingivalis* 33277 RNA as a positive control and nuclease-free water (Sigma-Aldrich, Dorset, UK) as a negative control. All RT-qPCR assays were performed in a LightCycler 480 Real-Time PCR (Roche Molecular Systems, West Sussex, UK). The thermocycling program was: i) step-1 (reverse transcription):10 min at 48 °C, ii) step-2 (cDNA amplification): 10 min at 95 °C (initial denaturation), 40 cycles of 30 s at 95 °C, 1 min at 60 °C. The increase in fluorescence was monitored during PCR amplification and all data was analysed using LightCycler 480 Software (Roche Molecular Systems, West Sussex, UK). The expression levels were normalised against 3 reference genes (*recA*, *glyA* and *groL*) for relative quantification using the ΔΔCT method [30].

Section 3: Enzyme-linked immunosorbent assay (ELISA) to quantify different anti-*P. gingivalis* antibody isotypes

High binding polystyrene 96-well plates were coated with *P. gingivalis*-purified lipopolysaccharide (InvivoGen, Toulouse, France) at 1µg/mL concentration and stored overnight at 4 °C. Thereafter, plates were blocked with 5 % bovine serum albumin in PBS at room temperature for 30 minutes. Each serum and saliva sample at 1:10 dilution was incubated in duplicate for 2 hours at room temperature. The bound serum IgG, IgM and IgA antibodies were detected by horseradish peroxidase-conjugated, anti-human IgG (A0170, Sigma-Aldrich, Dorset, UK) diluted at 1:30000, anti-human IgM (A6907, Sigma-Aldrich, Dorset, UK) diluted at 1:10000, and anti-human IgA (A18781, Thermo Fisher Scientific) at 1:2500 concentrations, respectively. Similarly, for the plate containing saliva samples, an anti-human IgA2 antibody (BT91-4005, Generon, Dublin, Ireland) was added at 1:1000 concentration. Samples were visualised spectrophotometrically at 492 nm using o-Phenylenediamine dihydrochloride (SIGMAFAST OPD, P9187 Sigma-Aldrich, Dorset, UK). The dilution factors for test sera, saliva samples and HRP-conjugated antibodies were optimised before the main experiment (data not shown). Spectrophotometry values were corrected for non-specific binding of the HRP-conjugated.

Supplementary Tables

Supplementary Table 1: The primer-probe sequences used to target and amplify 16S rRNA gene hypervariable regions of four periodontal bacteria: *Porphyromonas gingivalis, Tannerella forsythia*, *Aggregatibacter actinomycetemcomitans* and *Prevotella intermedia* in qPCR assays

16S ribosomal RNA gene	Primers 5'→ 3'	Probe 5'→ 3'	Amplicon length (bp)
1. *Porphyromonas gingivalis* ATCC 33277, GenBank accession: L16492.1, Region: 1-1474	Forward: CGTGAAGGAAGACAGTCCTAAG<br>Reverse: CGGAGTTAGCCGATGCTTATT	[6FAM]TACGGGAATAACGGGCGATACGAGTA [TAM]	128
2. *Tannerella forsythia* ATCC 43037, GenBank accession: NR_040839.1, Region: 1-1466	Forward: CGGTGAAAGTTTGTCGCTTAAC<br>Reverse: GGAGTTCTGCGTGATCTCTATG	[6FAM]AAGTAGGCGGAATGCGTGGTGTAG[TAM]	130
3. *Aggregatibacter actinomycetemcomitans* ATCC *29523*, GenBank accession: M75038.1 Region: 1-1480	Forward: ACGGGAAACTGTCGCTAATAC<br>Reverse: CCCACCAACTACCTAATCACAC	[6FAM]GTAGAGTCGGGAGACGAAAGTGCG[TAM]	105
4. *Prevotella intermedia* ATCC 25611, GenBank accession: NR_113107.1, Region: 1-1380	Forward: CAGAGGGACGGTGTAATGTAAA<br>Reverse: CGCGATTACTAGCGAATCCA	[6FAM]ATCCAATCTTGAAAGCCGGTCCCA[TAM]	106

ATCC-American Type Culture Collection, bp-base pairs, 6FAM- reporter dye and TAM-quencher

Supplementary Table 2: Details of proteins encoded by the thirteen virulence genes targeted in this study and their respective biological functions that contribute to periodontitis

Virulence genes	Encoded proteins	Biological functions
kgp	Lys-gingipain	• has a potent proteolytic activity and accounts for 85% *P. gingivalis*-induced tissue breakdown [15];<br>• upregulates pro-inflammatory cytokines via TLR-2 pathway- IL1β, IL-6 and its receptor, IL-8, IL-12, IL-4, interferon-γ, tumour necrosis factor-α, and Intercellular adhesion molecular-1[15];<br>• functions as ligands to promote co-aggregation with other red-complex bacterial members- *T. denticola* and *T. forsythia* [15];<br>• cleaves human haemoglobin via transpeptidase activity to acquire iron and protoporphyrin IX, an absolute requirement for *P. gingivalis* growth and in turn becomes bloodborne [15].
rgpA	Gingipain R1	• degrades type I and IV collagens [15] and cleaves C3 molecule, potentially contributing to decreased bacterial opsonisation [15];<br>• captures the human complement inhibitor C4b-binding protein via hemagglutinin-adhesin domain and thereby, hindering deposition of the membrane attack complex and complement-mediated killing [15];<br>• upregulates pro-inflammatory cytokines via TLR-2 pathway- IL1β, IL-6 and its receptor, IL-8, IL-12, IL-4, interferon-γ, tumour necrosis factor-α, and Intercellular adhesion molecule-1[15].
fimA	Major fimbrium subunit FimA type-2	• enhances bacterial adhesion to multiple types of surfaces, such as the extracellular matrix, host cells and other bacteria- critical for biofilm formation [15];<br>• fimA genotypes II and IV are frequently detected in patients with periodontitis and have strong epithelial cell adhesion and invasion properties [15] and fimA type II genotype is most prevalent in coronary arterial atheromatous plaque samples from patients with periodontitis [15];<br>• activates gingival fibroblasts, monocyte and macrophages via TLR2 pathway to induce the production of TNF-α, IL-1α, IL-1β, and IL-6 cytokines [15];
bioF-3	8-amino-7-oxononanoate synthase sphingolipids	• stimulates human gingival fibroblasts via TLR-2 pathway to upregulate the production of pro-inflammatory cytokines-interleukin-1β (IL-1β), prostaglandin E2 [PGE2] and 6-keto-prostaglandin F2α) [15];<br>• causes endothelial cell apoptosis [15] and induces the receptor activator of nuclear factor-kappa-B ligand (RANKL) mediated osteoclastogenesis and thereby promoting tissue breakdown in periodontal disease [15].
prtH	PrtH protease	• causes detachment of epithelial cells and cytopathic activity arrest cells in their G2 phase [17] and increases the mitochondrial oxidative membrane potential in cells, leading to IL-8 production from detached fibroblast cells and neutrophil recruitment [17];<br>• *prtH* genotype levels are strongly associated with advanced periodontal attachment loss compared to periodontally-healthy subjects [17].
bspA	Surface antigen BspA	• involves in protein-protein interactions, signal transduction, bacterial adherence and invasion into epithelial cells [17];<br>• triggers TLR-2-dependent release of bone-resorbing pro-inflammatory cytokines from monocytes and chemokine IL-8 from gingival epithelial cells [17];<br>• binds to the extracellular matrix component fibronectin and the clotting factor fibrinogen [17];<br>• *T. forsythia* mutant defective in the expression of BspA protein showed significantly less bone loss as compared to mice infected with the wild-type strain [17].

siaHI	Exo-alpha-sialidase	• catalyses glycosidic linkages into oligosaccharides and proteoglycans for nutrients acquisition but this catalytic process, in turn, affects the functional integrity of periodontium and promotes periodontal disease progression [17];<br>• the glycosidase activity exposes protein epitopes for adherence and coaggregation by other bacteria [17];
prtP	Serine protease (Dentilisin)	• disrupts cell-cell junctions, degrade laminin (type VI collagen) and invade the epithelial monolayers' barrier[16] and degrades IL-1β, IL-6, tumour necrosis factor-alpha (TNF-α), and monocyte chemoattractant protein-1[16];<br>• interacts with *P. gingivalis*-fimbriae to promote co-aggregation with members of "red-complex bacteria" [16];<br>• *T. denticola* and *P. gingivalis* synergistically hydrolyse fibrinogen, thereby promoting vascular disruption, bleeding and inflammation and retarding tissue repair [16].
msp	Major outer sheath protein	• has a potent pore-forming cytotoxic activity towards periodontal ligament epithelial cells, causing cell detachment and apoptosis [16];<br>• blocks the IL-8 production required for neutrophil chemotaxis and phagocytic and thereby evade the host immune response [16];<br>• degrades host cell protease inhibitors and fibronectin [16];<br>• an immunodominant antigen of *T. denticola* that elicits a strong antibody response [16].
ltxA	Leukotoxin A	• the large pore-forming toxin that binds to the lymphocyte function-associated receptor 1 (LFA-1) and disrupts the membrane integrity of most of the immune cells, leading to cell death and immune evasion [18];<br>• activates neutrophil degranulation causing a massive release of lysosomal enzymes, net-like structures, and matrix metalloproteinases (MMPs) and induces apoptosis in lymphocytes [18];<br>• activates inflammasome complex in the monocytes/macrophages, including the cysteine proteinase caspase-1, which induces activation and secretion of the pro-inflammatory cytokines IL-1β and IL-18 [18];<br>• frequently detected with serotype b and JP2 isolates that are strongly associated with rapid periodontal attachment loss [18].
cdtB	Cytolethal distending toxin subunit B	• part of a tripartite complex comprised of subunits CdtA, CdtB, and CdtC, while CdtB protein is the active subunit. It has a potent type I DNase activity causing G2 phase cell arrest of a variety of proliferating cells, including epithelial cells, fibroblasts, human periodontal ligament cells, and lymphocytes, leading to cell apoptosis [18];<br>• targets and invades the immune responses by inducing apoptosis of non-proliferative monocytic cells and T lymphocytes [18];<br>• stimulates the production of pro-inflammatory cytokines by peripheral blood mononuclear cells, such as interferon (IFN)-γ, Interleukin (IL)-1β, IL-6, and IL-8; a virulence property potentially independent of the toxin's type I DNase activity [18];<br>• stimulates RANKL production by periodontal fibroblasts and periodontal ligament cells as well as T-cells, leading to bone loss [18].
clpB	Chaperone protein ClpB	• ATP-dependent chaperone protein that is involved in the quorum sensing system of periodontal microbial biofilm and aiding coaggregation of *P. gingivalis* by upregulating its virulence factors, such as iron uptake, flagellar protein synthesis and gingipain production [29].
dnaK	Chaperone protein DnaK	• stress or heat shock protein (hsp70) is up-regulated during infection and involved in conserved cellular processes, such as protein-folding reactions and the assembly/disassembly of protein complexes [29];<br>• anti-hsp70 antibodies cross-react with HSPs expressed on the endothelial cells and thereby contribute to the atherosclerosis process [29].

Supplementary Table 3: The primer and probe sequences used to target and amplify thirteen genes (1-13) that encode the major virulence factors of 5 primary periodontal bacteria. Primer and probe sequences of three (14-16) reference genes used for normalisation in ΔΔCt method

Targeted genes	Primers 5'→ 3'	Probe 5'→ 3'	Amplicon length (bp)
**Virulence genes of five periodontal bacteria**			
1. kgp, *Porphyromonas gingivalis strain W83, GenBank accession:CP025932, Region:1939820-1940915*	Forward: GGAGACCAAAGGTGGTACTTTC<br>Reverse: GCACAGCAATCAACTTCCTAAC	[6FAM]GGTGAGGTTGGTTCTCCCGAAGTG[Eclipse® Dark Quencher]	139
2. rgpA *Porphyromonas gingivalis strain W83, GeneBank accession:CP025932, Region: 2119432-2125762*	Forward: CTTCCACCACCTTCGCTTATAG<br>Reverse: GGACCGACGAAAGAAGATGATTA	[6FAM]TTCAGTTCCATCACCGCTACCCAT[Eclipse® Dark Quencher]	143
3. fimA *Porphyromonas gingivalis, GeneBank accession: AB195793, Region:103-1257*	Forward: ATGTTGACTGGTTGGGAAGAG<br>Reverse: CCTGCATTCTGAGCGTATGT	[6FAM]AGCCTTCCAATAATGCTCCACAAGGT[Eclipse® Dark Quencher]	141
4. bioF-3 *Porphyromonas gingivalis strain W83, GeneBank accession:AE015924, Region:1868926-1870113*	Forward: CGACACCTATTATCCCGCTTT<br>Reverse: GCACAGCAATCAACTTCCTAAC	[HEX]TCCGCAACAACGAGAAGACATTCCA[Eclipse® Dark Quencher]	97
5. prtH *Tannerella forsythia strain ATCC 43037,GeneBank accession: AB001892, Region:193-1464*	Forward: GGCTATCCCACTTCTTTCACTC<br>Reverse: CACCGCATCCATTCCGTATAA	[6FAM]TGGTGTAAGTGTTTGGATGTATGAAGAGGC[Eclipse® Dark Quencher]	117
6. bspA *Tannerella forsythia strain ATCC 43037,GeneBank accession: AF054892, Region: 112-3357*	Forward: TCCCAAAGACGCGGATATCA<br>Reverse: ACGGTCGCGATGTCATTGT	[HEX]CCGCGACGTGAAATGGTATTCCTC[Eclipse® Dark Quencher]	66
7. siaHI *Tannerella forsythia strain ATCC 43037,GeneBank accession: AY069941, Region: 193-1590*	Forward: GGGCGACCAGTATGATAACTTC<br>Reverse: TTGGCGCAACCGTCTATC	[Cy®5]TGGAGAACGGGATTCATCTGCACA[DDQII]	93
8. prtP *Treponema denticola strain ATCC 35405, GeneBank accession:D83264, Region:1515-3683*	Forward: AAGCAAGGCTCCGAATCAA<br>Reverse: GCCTCAGGGTCATCCAAATAA	[6FAM]ACGATAGCCCTGTAGACCCTTCCA[Eclipse® Dark Quencher]	96

**9. *msp***<br>*Treponema denticola strain ATCC 35405, GeneBank accession:KJ671481, Region:455610- 457241*	Forward: CTGTTGACGGTCTTGCTCTAA<br>Reverse: ACCGAAATAGGCACCAAGAG	[HEX]TAGGCACGGATTCAAAGGTCGCTT[Eclipse® Dark Quencher]	128
10. ltxA<br>*Aggregatibacter actinomycetemcomitans strain ATCC 29524, GeneBank accession: KY965314, Region: 636-3802*	Forward: CAGCACAGAAGTTAGGGATTGA<br>Reverse: CTCGTTCGGTAAGACCAAGTAAT	[Cy®5]AAGGGAAAGACGGCCCAGCATTAA[DDQII]	108
11. cdtB<br>*Aggregatibacter actinomycetemcomitans strain serotype a, GeneBank accession: AY366473, Region:11-862*	Forward: GTAGGTATCCGCATTGGTACTG<br>Reverse: GGTGATGATGGTGATGAGGTAAA	[6FAM]ACAGGTGGTTCTGATGCGGTAAGT[Eclipse® Dark Quencher]	122
12. clpB<br>*Prevotella intermedia strain 17, GeneBank accession: CP019302, Region:1688488-1691076*	Forward: GAGAGCAGCCATAACAGAAC<br>Reverse: CCAAGTTGCGTGCGTATTTC	[HEX]ACACAAAGCGGCGACGAGAACTAT[Eclipse® Dark Quencher]	99
13. dnaK<br>*Prevotella intermedia strain strain 17, GeneBank accession: CP019302, Region: 779640-781541*	Forward: GATGAAGAAGACCGCTGAAGA<br>Reverse: CGCTGTGAGTCAGAGAAGTAAG	[Cy®5]TGGACAAGAGGTAACAGACGCAGT[DDQII]	87
**Reference genes used for normalisation in ΔΔCt method**			
14. RecA<br>*Porphyromonas gingivalis ATCC 33277, GenBank accession: CP025930, Region: 1178008-1179030*	Forward: TTGGGCGTCAATGTGGATAA<br>Reverse: GACAGCAGAAGAGCGAATCA	[6FAM]CGGCGATTTCCAAAGCCTGTTCAC[TAM]	137
15. glyA<br>*Porphyromonas gingivalis ATCC 33277, GeneBank accession: CP025930, Region:46617- 47897*	Forward: AGGCTATGGGTAGCTGTATGA<br>Reverse: GATACGGTCGATGGCGATTT	[HEX]TATGCCGAAGGTTATCCCGGCAAA[Eclipse® Dark Quencher]	113
16. groL<br>*Porphyromonas gingivalis ATCC 33277, GeneBank accession: CP025930, Region:1649350- 1650987*	Forward: GTGAGCGTAGCGAAAGAGATAG<br>Reverse: CATTGGTCTTGGAGGCTACTT	[Cy®5]ATTGGAGTGCCCGTTCGAGAACAT[DDQII]	88

ATCC-American Type Culture Collection, bp-base pairs, 6FAM, HEX, Cy®5- reporter dyes and TAM, Eclipse® Dark Quencher, DDQII-quenchers

Supplementary Figures

Supplementary Figure 1: Strengthening the reporting of observational studies in epidemiology (STROBE) checklist

STROBE Statement—checklist of items that should be included in reports of observational studies

	Item No	Recommendation	Page No
**Title and abstract**	1	(*a*) Indicate the study's design with a commonly used term in the title or the abstract	1
		(*b*) Provide in the abstract an informative and balanced summary of what was done and what was found	2
**Introduction**			
Background/rationale	2	Explain the scientific background and rationale for the investigation being reported	3
Objectives	3	State specific objectives, including any prespecified hypotheses	3
**Methods**			
Study design	4	Present key elements of study design early in the paper	6
Setting	5	Describe the setting, locations, and relevant dates, including periods of recruitment, exposure, follow-up, and data collection	6
Participants	6	(*a*) *Cross-sectional study*—Give the eligibility criteria, and the sources and methods of selection of participants	6
Variables	7	Clearly define all outcomes, exposures, predictors, potential confounders, and effect modifiers. Give diagnostic criteria, if applicable	6, 7, 8
Data sources/ measurement	8*	For each variable of interest, give sources of data and details of methods of assessment (measurement). Describe comparability of assessment methods if there is more than one group	*6, 7, 8*
Bias	9	Describe any efforts to address potential sources of bias	6, 7, 8
Study size	10	Explain how the study size was arrived at	6
Quantitative variables	11	Explain how quantitative variables were handled in the analyses. If applicable, describe which groupings were chosen and why	6, 7, 8
Statistical methods	12	(*a*) Describe all statistical methods, including those used to control for confounding	8
		(*b*) Describe any methods used to examine subgroups and interactions	8
		(*c*) Explain how missing data were addressed	N/A
		(*d*) *Cross-sectional study*—If applicable, describe analytical methods taking account of sampling strategy	8
		(*e*) Describe any sensitivity analyses	8
**Results**			
Participants	13*	(a) Report numbers of individuals at each stage of study—eg numbers potentially eligible, examined for eligibility, confirmed eligible, included in the study, completing follow-up, and analysed	3
		(b) Give reasons for non-participation at each stage	12
		(c) Consider use of a flow diagram	12
Descriptive data	14*	(a) Give characteristics of study participants (eg demographic, clinical, social) and information on exposures and potential confounders	3
		(b) Indicate number of participants with missing data for each variable of interest	N/A
		(c) *Cohort study*—Summarise follow-up time (eg, average and total amount)	N/A
Outcome data	15*	*Cross-sectional study*—Report numbers of outcome events or summary measures	3, 4
Main results	16	(*a*) Give unadjusted estimates and, if applicable, confounder-adjusted estimates and their precision (eg, 95% confidence interval). Make clear which confounders were adjusted for and why they were included	3, 4
		(*b*) Report category boundaries when continuous variables were categorized	3, 4
		(*c*) If relevant, consider translating estimates of relative risk into absolute risk for a meaningful time period	3, 4
Other analyses	17	Report other analyses done—eg analyses of subgroups and interactions, and sensitivity analyses	3, 4
**Discussion**			
Key results	18	Summarise key results with reference to study objectives	4
Limitations	19	Discuss limitations of the study, taking into account sources of potential bias or imprecision. Discuss both direction and magnitude of any potential bias	6
Interpretation	20	Give a cautious overall interpretation of results considering objectives, limitations, multiplicity of analyses, results from similar studies, and other relevant evidence	4, 5. 6
Generalisability	21	Discuss the generalisability (external validity) of the study results	4, 5. 6
**Other information**			
Funding	22	Give the source of funding and the role of the funders for the present study and, if applicable, for the original study on which the present article is based	11

Supplementary Figure 2: Illustration detailing periodontal pocket anatomy and periodontal measurements recorded for calculation of periodontal inflamed surface area (PISA)

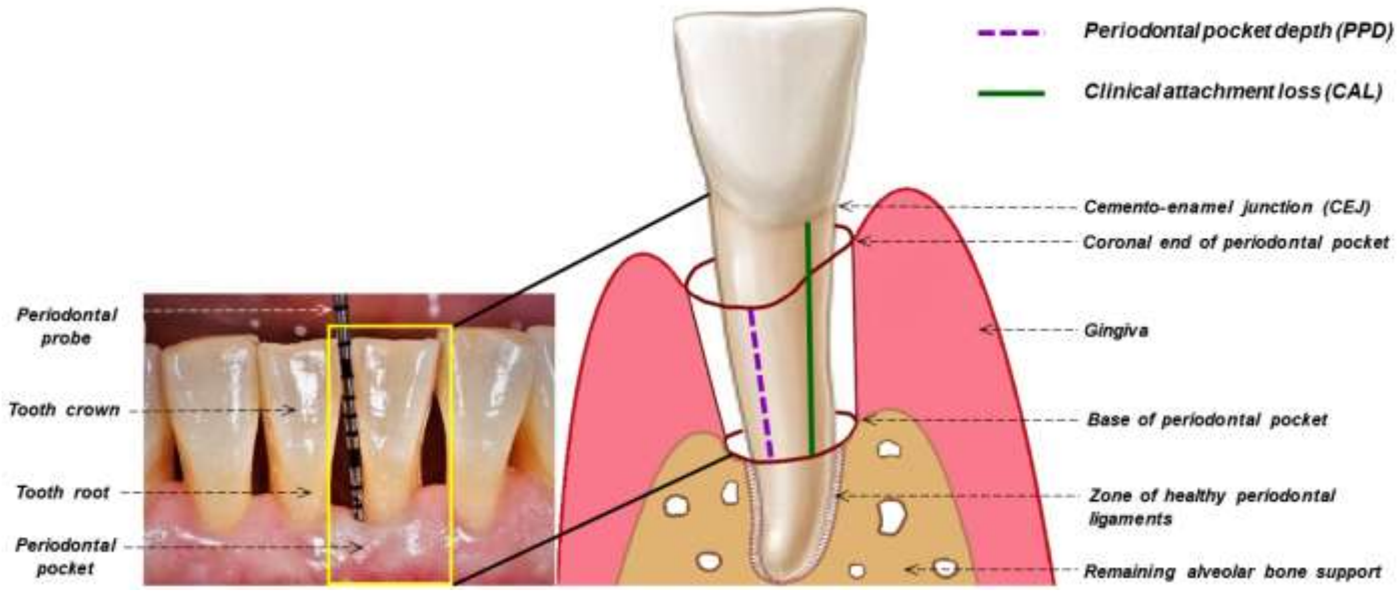

Periodontal pocket depth (PPD), clinical attachment loss (CAL) and the number of bleeding on probing (BoP)-positive sites were entered into the excel spreadsheet containing inbuilt formulas to calculate periodontal inflamed surface area (PISA). http://www.parsprototo.info [26]

Supplementary Figure 3: Standard curves generated for the reference strains of primary periodontal pathobiont species: *Porphyromonas gingivalis, Tannerella forsythia, Aggregatibacter actinomycetemcomitans* and *Prevotella intermedia*, using qPCR assays

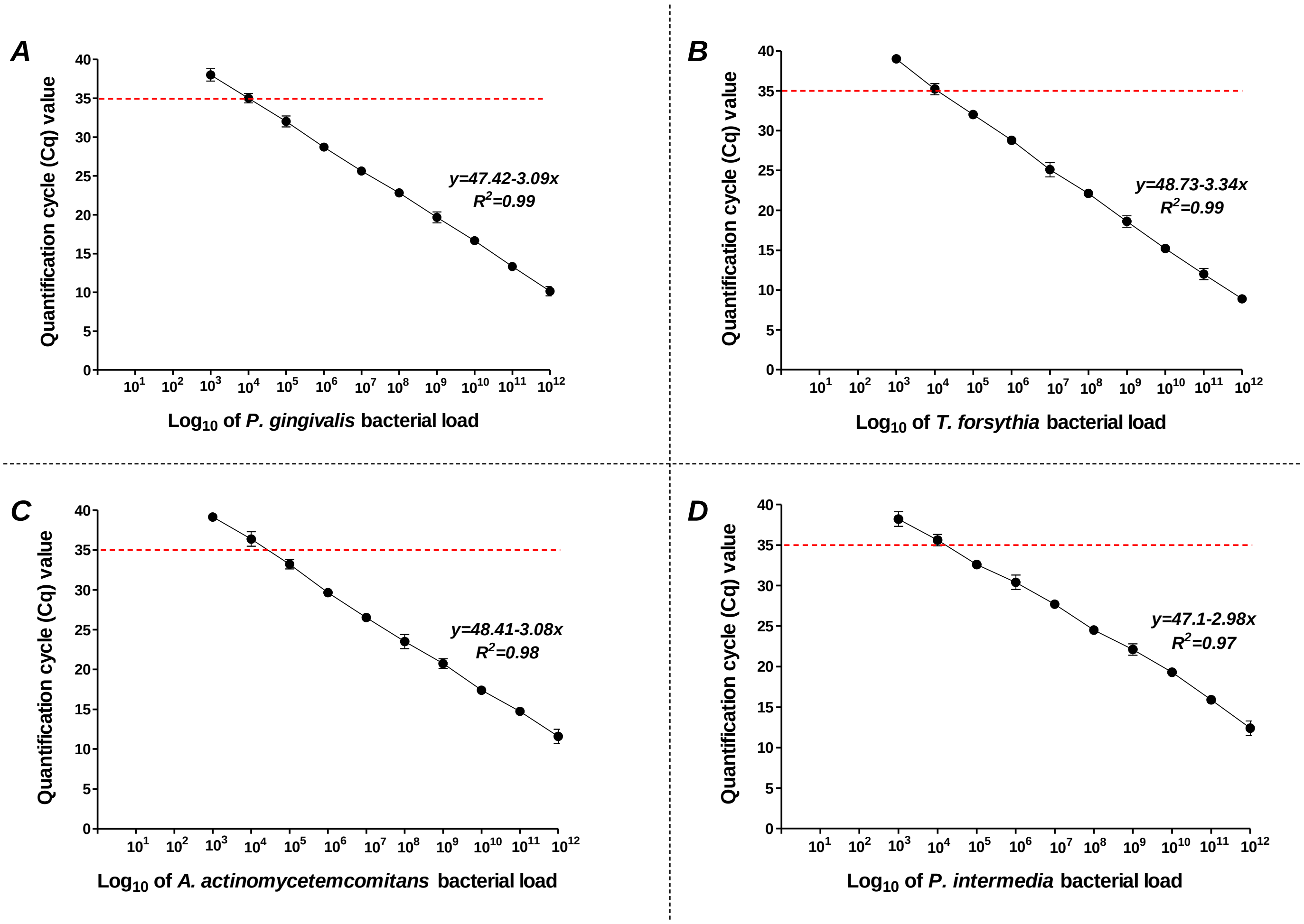

The $\log_{10}$ transformed serially diluted bacterial loads and the corresponding quantification cycle (Cq) values generated in qPCR assay plotted for: A) *P. gingivalis*, B) *T. forsythia*, C) *A. actinomycetemcomitans* and D) *P. intermedia.* The linear regression equations generated by each standard curve were used to calculate the bacterial load of the respective species in the tested samples. R^2 = squared correlation coefficient. The red dotted red lines in each plot represent the upper cut-off limit of 35 Cq, set for bacterial detection.
